# An Approach for Simulation of the Muscle Force Modeling It by Summation of Motor Unit Contraction Forces

**DOI:** 10.1155/2013/625427

**Published:** 2013-10-03

**Authors:** Rositsa Raikova, Hristo Aladjov, Jan Celichowski, Piotr Krutki

**Affiliations:** ^1^Institute of Biophysics and Biomedical Engineering, Bulgarian Academy of Sciences, Academy G. Bonchev Street, Block 105, 1113 Sofia, Bulgaria; ^2^Department of Neurobiology, University School of Physical Education, 27/39 Krolowej Jadwigi St., 61-871 Poznan, Poland

## Abstract

Muscle force is due to the cumulative effect of repetitively contracting motor units (MUs). To simulate the contribution of each MU to whole muscle force, an approach implemented in a novel computer program is proposed. The individual contraction of an MU (the twitch) is modeled by a 6-parameter analytical function previously proposed; the force of one MU is a sum of its contractions due to an applied stimulation pattern, and the muscle force is the sum of the active MUs. The number of MUs, the number of slow, fast-fatigue-resistant, and fast-fatigable MUs, and their six parameters as well as a file with stimulation patterns for each MU are inputs for the developed software. Different muscles and different firing patterns can be simulated changing the input data. The functionality of the program is illustrated with a model consisting of 30 MUs of rat medial gastrocnemius muscle. The twitches of these MUs were experimentally measured and modeled. The forces of the MUs and of the whole muscle were simulated using different stimulation patterns that included different regular, irregular, synchronous, and asynchronous firing patterns of MUs. The size principle of MUs for recruitment and derecruitment was also demonstrated using different stimulation paradigms.

## 1. Introduction

 The force of a skeletal muscle is an accumulation of forces generated by active motor units belonging to this muscle. A motor unit (MU) is a motor neuron and all the muscle fibers innervated by its axon. The MU is the smallest functional element of the neuromuscular system. Motor units develop forces in response to trains of motoneuronal action potentials transmitted to the muscle fibers by motor axons. The central nervous system controls the muscle force by two basic mechanisms: (1) rate coding alters interpulse intervals (IPIs) between successive action potentials, which is measured as discharge rate and (2) recruitment-derecruitment processes regulate the number of active MUs [1–5]. Since it is very difficult to study these processes using *in vivo* experiments, the modeling of muscle force as a result of different types of MUs' activity patterns can enhance our understanding of force control processes. Several muscle models consisting of MUs were proposed [6–10]. The most complex and frequently used model in various modifications appears to be the one proposed by the group of Fuglevand [6, 11]. 

 Several elements of physiological knowledge should be taken into account with respect to the evaluation of a realistic muscle model. The force developed by one MU in response to a single stimulus (the twitch) has often been modeled by an analytical function, which accounts for only two parameters: the maximal twitch force and the contraction time. Fuglevand et al. [6] model the MU twitch force using a power function which results in a fixed relationship between the maximum twitch force and the contraction time. The distribution of MUs based on maximum twitch force and contraction time within a modeled MUs' pool has been approximated using general exponential equations based on experimental findings [6, 9, 12, 13]. However, it was shown in [14] that the contraction time and the maximal force amplitude of an MU twitch are insufficient to describe the considerable variability of the twitch forms in a real muscle. Moreover, different muscles (slow, fast) have MUs with different dynamics of force development and with variable twitch parameters which do not fulfill a strict dependence between maximal twitch force and contraction time.

 Despite the fact that some models incorporate fast and slow MUs [15], their unique contributions to whole muscle force have not been fully developed. Specifically, this refers to the prolonged relaxation time and the enhanced effectiveness of summation of successive contractions for slow MUs and higher maximal forces and shorter twitch duration for fast MUs. Furthermore, models have focused on recruitment and not on derecruitment of MUs' activity. The recruitment order in models is based on the size principle which is easily approximated by exponential equations [16–20]. These models did not consider the specificity of the motor task, which could have modified the acknowledged recruitment order (low force, slow twitch MUs are commonly recruited before high force, fast twitch MUs), as reported by some authors [10, 21–25]. The aim of the particular motor task and the afferent feedback loops are important to understand how the central nervous system solves the highly indeterminate problem due to the infinite number of combinations of motoneuronal firing patterns and MUs' forces which can generate the appropriate muscle force in order to perform the planned movement. 

 We have proposed a muscle model composed of a number of MUs with variable properties based on MUs' twitches [26], and this model was used for investigation of human elbow flexion/extension movements [26–29]. The twitches have been approximated with a 4-parametric function, and their parameters, as well as the number of MUs for human muscles, have been estimated based on the literature data. Contrary to all other models, the discharge rate of the MUs has not been preset but was matched by using a hierarchical genetic algorithm and multiple optimizations during the whole time period of a given motor task. In general, this means that the algorithm chooses the respective firing of all MUs so that some criteria connected to the modeled motor task can be fulfilled. However, Henneman's size principle has not been included in the software. This limitation can be avoided in the future by adding a new program module. A disadvantage of such approach is the large computation time associated with using a realistic number of MUs. Moreover, the time required to complete the simulation increases with the duration of the motor task. 

 This paper has four main goals: (1) to present a more realistic muscle model taking into account the variability in MU twitch shapes and providing the possibility to assign individual firing patterns for each of MU within the muscle; (2) to develop a simple, user friendly computer program for muscle model that can easily be adapted for different mixtures of MUs based on muscle fiber type; (3) to test the model using real twitches obtained from experimental recordings on the rat medial gastrocnemius muscle; and (4) to investigate the forces developed by individual MUs and by the whole muscle using different firing patterns: stimulation rates at constant IPIs, random stimulation patterns with variable IPIs, and patterns for which the order of MUs' recruitments follows the size principle.

## 2. Model and Software

**Figure 1:**
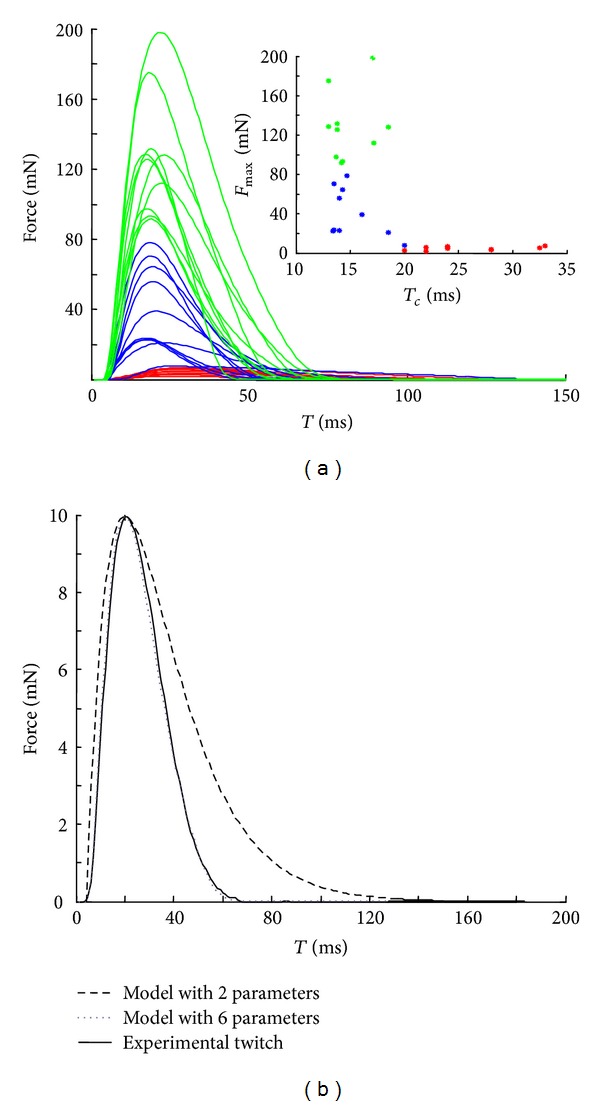
Twitches of the rat medial gastrocnemius muscle. (a) The models of the experimentally measured twitch forces of the chosen 30 MUs (red: *S* MUs, blue: FR MUs, and green: FF MUs). In the upper right corner, the dependence between *F*_max⁡_ and *T*_*c*_ is presented for all MUs (the same colors are used for the three MU types). (b) Comparison between one experimental twitch and its two models by using the 6-parameter function from Raikova et al. [14] and the 2-parameter function from Fuglevand et al. [6].

 The model and the software were tested using 30 MUs, with twitches (forces evoked by the application of one impulse) that were measured during *in vivo* experiments on the rat medial gastrocnemius muscle. The experimental procedure has been described elsewhere [30, 31]. Ten of the chosen MUs were slow (*S*), 10 were fast-fatigue resistant (FR), and 10 were fast fatigable (FF). The relationship between the maximal force and the contraction time for these MUs followed an inverse power function (Figure 1(a)) and was in agreement with the one accepted in Fuglevand et al. [6]. 

For each MU, the experimental twitch shape was modeled by the 6 parameter analytical function proposed in Raikova et al. [14], which was tested and verified on a large group of twitches recorded experimentally. These 6 parameters were *F*_max⁡_: maximal twitch force; *T*_lead_: lead time, the time between the stimulus and the start of force development; *T*_hc_: half-contraction time, the time from the start of the contraction to the time where the force reaches one half of its maximal value; *T*_*c*_: contraction time, the time from the start of the contraction to the time when the force reaches its maximal value; *T*_hr_: half-relaxation time, the time from the start of the contraction to the moment where during the relaxation phase the MU force decreases to its half maximal value; *T*_tot_: the duration of the twitch (see Figure 2(a) in Raikova et al. [14]). This 6 parameter analytical function was deemed suitable to describe with a high degree of accuracy the twitch forms of numerous experimentally recorded MUs (see Figure 4 in Raikova et al. [14]). The analytical twitch models of the currently chosen MUs are shown in Figure 1(a). The simulated twitch force produced by the model used in [6] with only 2-parameters (maximal twitch force and contraction time) is presented in Figure 1(b) for comparison. It is important to emphasize that this calculated force is shifted in time with the lead time experimentally measured for the same MU. It can be seen in Figure 1(b) that the 2 parameter twitch model is not able to follow the shape of the force curve very well, especially the relaxation phase.

**Figure 2:**
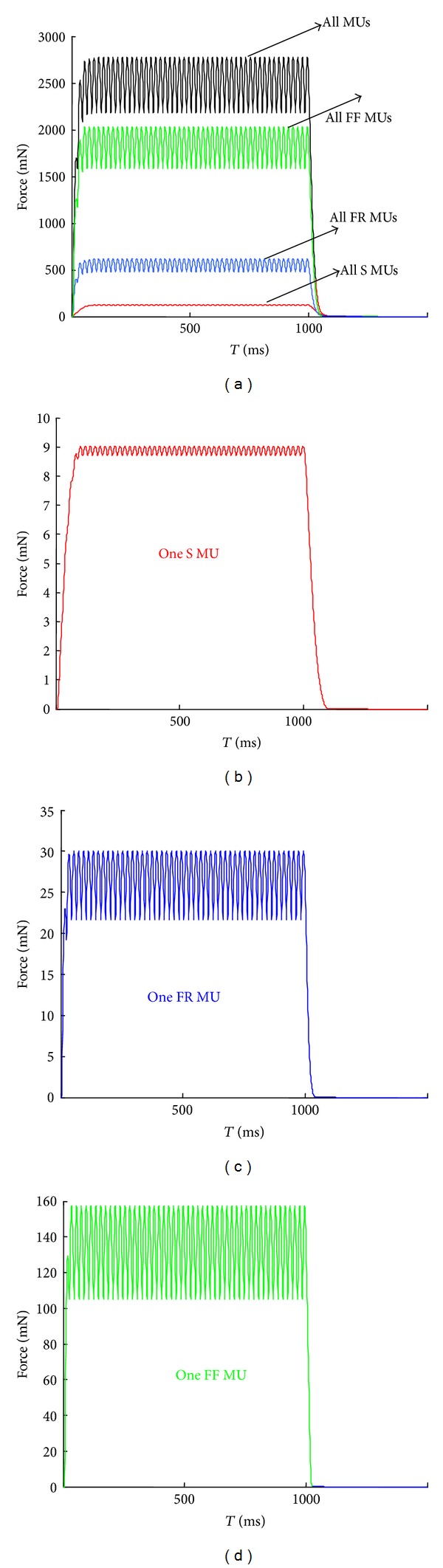
Simulation of the muscle and MUs' forces using the same regular pattern for all MUs. 50 stimuli with IPI of 20 ms were applied to each MU. (a) The whole muscle force is in black, the sum of forces of all slow MUs is in red, the sum of forces of all FR MUs is in blue, and the sum of all FF MUs is in green. (b)–(d) present the simulated forces of one *S*, one FR, and one FF MU, respectively, after application of the same firing pattern. Note that the force scales are different for the four plots.

 It is well known that when MUs are activated by series of stimuli, the IPIs determine whether nonsummating twitches, unfused tetani with different peak forces or fused tetani, can be evoked. To model all these possibilities of force regulation, it was accepted in the present model that each individual stimulus evokes one contractile response (twitch-like force), and the application of a series of stimuli evokes a train of responses, which are mathematically summated with a simplification that all twitch-shape responses are equal for a particular MU. The total muscle force output was obtained by summation of forces generated by all active MUs (Figure 2).

Custom software written in MATLAB was developed to calculate MUs and muscle forces as a result of the application of different stimulation patterns. The inputs were taken from a text file (*datatw.txt*) with *N* rows and 6 columns. The total number of rows (*N*) corresponds to the number of MUs constituting the muscle, which can be different than the 30 used in the present study. Each column corresponds to one of the 6 parameter values that describe the twitch profile for the particular MU in the following order: *T*_lead_, *T*_hc_, *T*_*c*_, *T*_hr_, *T*_tot_, and *F*_max⁡_. A text file (*impulses.txt*) with *N* columns and *k* rows contains data for the stimulation of all MUs. The variable *k* is the maximal number of stimuli which are applied for the MUs. Additional input parameters were *N*1, *N*2, and *N*3, which are the respective numbers of *S*, FR, and FF MUs; hence, *N* = *N*1 + *N*2 + *N*3. The last input parameter was the duration of the simulation, *T*_duration_. The MUs in the *datatw.txt* file were arranged according to the increasing twitch force amplitude within the respective group. The first *N*1 rows contain the slow MUs, next *N*2 rows: the FR MUs, and next *N*3 rows: the FF MUs. There were two variants for constructing *impulses.txt* file. In the first variation, the *i*th column contained the time moments of the successive stimuli of the *i*th MU. In the second variation, the first row consisted of constants equal to the time delay for recruitment of the respective MU, and the values downwards in the column were the consecutive IPIs of the respective MU firing (in this case the number of rows is *k* + 1). The text file with data for IPIs could be generated by a module in the software or could be downloaded as a previously prepared file in a text file or in *Excel* format sheet. The outputs of the model were the calculated forces of each MU, the calculated forces of all *S*, all FR, all FF MUs, and the calculated whole muscle force and, respectively, their graphics.

## 3. Results

 The methodology was demonstrated by simulation of a muscle model consisting of 30 MUs whose twitches were shown in Figure 1(a). The MUs were stimulated by application of several patterns: (1) regular synchronous firing: the IPIs were equal and constant for all MUs; (2) regular asynchronous firing: the IPIs were equal for all MUs, but for each MU they were shifted in time by random chosen constants; (3) irregular synchronous firing: for each MU one and the same pattern of IPIs was applied, and this pattern consisted of nonequal constants, randomly generated with a given mean value; (4) irregular asynchronous firing: for each MU an individual pattern of variable IPIs was generated with different mean frequencies (in a range from 10 Hz to 100 Hz, i.e., IPIs were from 100 ms to 10 ms); (5) irregular asynchronous firing with mean IPIs individually calculated for each MU as 1.25 times *T*_*c*_, which resulted in the generation of unfused tetani. The MU frequencies for irregular asynchronous firing had the following ranges: 24.2–40.0 Hz for *S* MUs, 42.1–59.7 Hz for FR MUs, and 43.2–61.5 Hz for FF MUs. Such frequencies resemble those observed for rat hind limb muscles in physiological conditions during natural movements [32].

Synchronous firing was defined in a way that all active MUs had the same firing pattern. Asynchronous firing was defined as some or all MUs having different firing patterns with different mean firing rates. The IPIs for the irregular stimulation patterns were randomly generated. In this case, the variability of MU firing rate ranged between 50% and 150% of the mean value of the IPIs for a given mean firing rate. Finally, the size principle of MUs' activation [17, 18] was also simulated. The recruitment order was modeled by setting different, increasing constants in the first row of the file *impulses.txt*, which resulted in activation of successive MUs according to their increasing forces. The derecruitment process was modeled in a reverse order, by determining the number of IPIs of the respective MUs, so that the last impulse was near at a preliminary chosen time point. 

**Figure 3:**
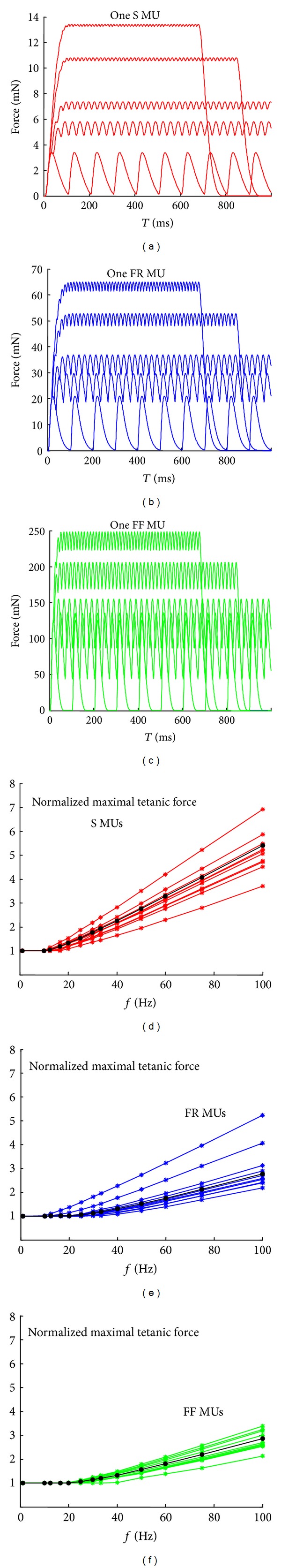
Main characteristics of the simulated muscle model: (a)–(c) representative tetanic forces of one *S*, one FR, and one FF MU, obtained by application of 50 pulses with IPIs of 100, 33.3, 25, 16.6, and 10 ms (i.e., the frequencies of 10, 30, 40, 60 and 100 Hz from below to upwards), respectively. Note that the time scale is cut to 1000 ms for better viewing; (d)–(f) relationship between the normalized maximal tetanic force and the firing frequency for each of the MUs. The consecutive points correspond to the following frequencies: 1, 10, 12.5, 16.6, 20, 25, 30, 33.3, 40, 50, 60, 75, and 100 Hz. Black curves and points represent the simulated forces of the whole group of *S*, FR, and FF MUs. The normalization for the figures (d), (e), and (f) is made individually for each MU with respect to the peak twitch force of the current MU (or the three groups of MUs), obtained for impulsation with 1 Hz.

 Synchronous discharges of all MUs in a muscle with constant IPIs may be considered as a model of whole muscle activity evoked by constant-frequency stimulation of a muscle nerve, frequently applied in physiological experiments [31, 33–36]. The plots in Figure 3 show that for each MU type the increase in frequency from 10 Hz to 100 Hz (i.e., the IPIs from 100 ms to 10 ms, resp.) resulted in increased peak of the tetanic forces; however, this increase was different for slow (Figures 3(a) and 3(d)) versus fast MUs (Figures 3(b), 3(c), 3(e), and 3(f)). Moreover, for slow units more fused tetanic force curves were observed (Figure 3(a) versus Figures 3(b) and 3(c)). 

 The calculated force-frequency relationships (Figures 3(d), 3(e) and 3(f)), within a frequency range corresponding to unfused tetani of particular MUs or of the whole muscle, were nearly linear. The differences between tetanic force curves of individual MUs are due to their twitch parameters. Changing the applied synchronous IPIs from 100 ms to 10 ms, the maximal total muscle force increased 2.93 times, the sum of forces of all FF MUs increased 2.86 times (Figure 3(f)), and the sum of forces of all FR MUs increased 2.77 times (Figure 3(e)), while the sum of forces of all slow MUs increased 5.4 times (even for one S MU this value reaches 6.91) (Figure 3(d)). 

**Figure 4:**
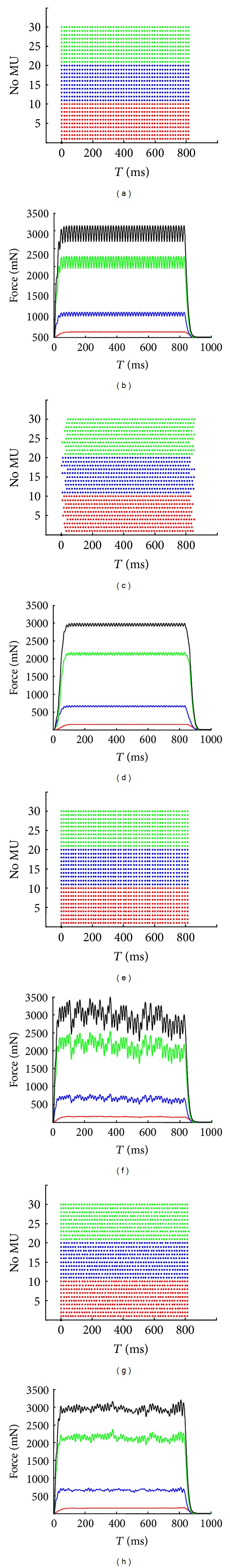
Comparison between synchronous and asynchronous and regular and irregular impulsation patterns for the simulated muscle model. 50 stimuli are applied to each MU. (a), (c), (e), and (g) show the impulsation patterns. (b), (d), (f), and (h) show the respective calculated forces. (a) and (c) show the respective synchronous and asynchronous patterns of stimuli at equal IPIs, presented for each MU as row of dots in the time scale. The frequency in both cases is 60 Hz (equal IPIs of 16.6 ms). The asynchronous pattern (c) is modeled by time shifting of the first spike of each MU with randomly chosen constants, from 0 to 40 ms. (b) and (d) show the calculated forces of *S* (red), FR (blue), and FF MUs (green), as well as of the whole muscle (black) for the respective impulsation patterns given in (a) and (c). (e) and (g) show the respective synchronous and asynchronous irregular patterns of stimuli, presented as dots. The IPIs are randomly generated with mean value of 16.6 ms (mean frequency of 60 Hz). The same irregular synchronous pattern is applied for all MUs in (e). The irregular patterns in (g) are different for each MU. (f) and (h) show the calculated forces of *S* (red), FR (blue), and FF MUs (green) as well as of the whole muscle (black) for the respective patterns given in (e) and (g).

 In general, regular, constant-frequency stimulation evoked uniform force curves, independently of whether they were synchronous or asynchronous (Figures 4(b) and 4(d)). The addition of slight desynchronization (i.e., assuming that the time moments of pulses in series of some MUs do not coincide) led to more fused tetanic curves of force, either of the whole muscle or of the groups of MUs, and produced lower maximal forces in comparison to synchronous firing patterns. This result is apparently due to the fact that the peaks of the individual twitches do not coincide when pulses are shifted in time.

Irregular, random impulses applied synchronously to all 30 MUs evoked visible oscillations of the whole muscle force (Figure 4(f)). However, when different random patterns (i.e., irregular and asynchronous impulsation) were applied to MUs, the whole muscle force curves were smoother (Figure 4(h) versus Figure 4(f), black lines). This observation can mainly be attributed to fast MUs force production, since the whole muscle force was predominantly determined by the strongest FF MUs (green line in Figure 4(h)). However, cumulative forces generated by all MU types became less variable. 

**Figure 5:**
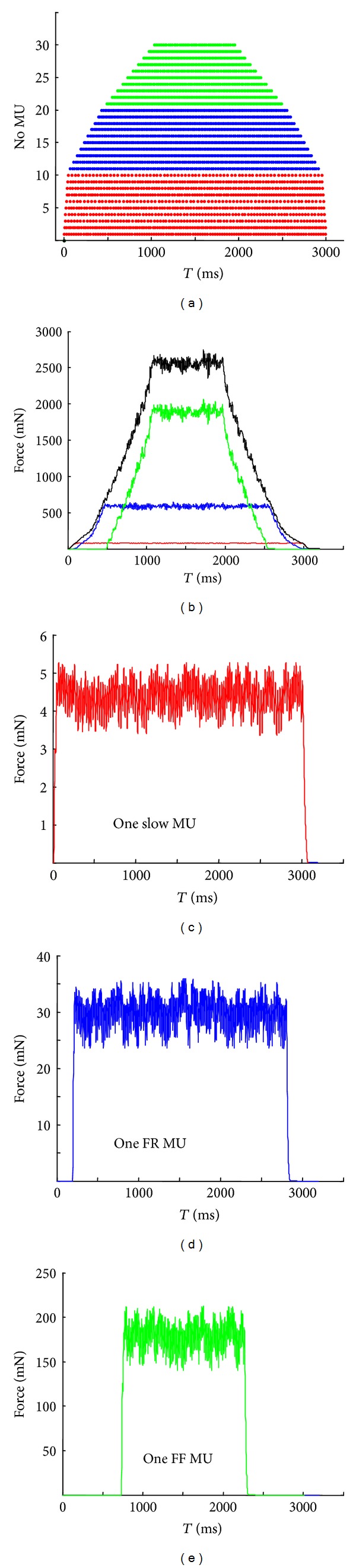
An example of stimulation applied to MUs respecting the size principle. (a) Patterns applied to the MUs, presented as dots in the time scale, each row corresponds to one MU, and patterns for each MU are different (randomly generated with different mean IPIs, from 16.2 ms to 40.90 ms, depending on the contraction time of the respective MU; for group of *S* MUs mean IPI is 30.53 ms; for group of FR MUs mean IPI is 19.22 ms; for group of FF MUs mean IPI is 18.68 ms); (b) the calculated forces of the whole muscle (black) and of the three groups of MUs: FF (green), FR (blue), and *S* (red), for the respective impulsation patterns given in (a); (c) forces calculated for one of the slow MUs (mean IPI = 25.1 ms); (d) force calculated for one of the FR MUs (mean IPI = 16.7 ms); (e) force calculated for one of the FF MUs (mean IPI = 23.33 ms). Note that the force scales are different.

The final step of the simulation concerned variable patterns of impulses for particular MUs during steady state of the muscle (these patterns were related to the *T*_*c*_ of each MU), when MUs were recruited and derecruited according to the size principle. The increase of the slope of the initial force was modeled by progressively increasing of the time moments of application of the first stimulus for each MU. The order of recruitment was from the weakest to the strongest MU (Figure 5(a)). After a period of activity, the MUs were derecruited in an order reverse to the recruitment. This type of firing pattern leads to a longer work time of the slow MUs in comparison to the fast ones, especially in the case of FF MUs (Figure 5(c) versus Figure 5(e)). The chosen mean frequencies during muscle steady state (plateaus in Figure 5(b)) produced a similar fusion of force profiles of all MUs.

## 4. Discussion

The aim of the paper was to develop and to present a simple, general tool for the simulation and investigation of muscle force generation and control. The muscle is composed of three types of MUs (*S*, FR, and FF), and the numbers of each type of MUs can be specified by the user. In this way different muscles can be modelled. The force that one MU can develop due to the application of one stimulus (the twitch) is determined by a 6 parameter function. The analytical function of the twitch form has been successfully tested on high number of MUs recorded during physiological experiments [14, 37]. These 6 parameters can be determined by the user to construct muscles with different MU compositions. The firing of all MUs can be also given by filling in a text or *Excel* file with time points of each stimulus. Thus different stimulation patterns for each MU can be applied. 

The approach and the custom-made software were demonstrated using 30 MUs derived from the rat medial gastrocnemius muscle, based on twitches measured during *in vivo* experiments. The simulated muscle was chosen to have an equal number of *S*, FR, and FF MUs since different muscles have different proportions of these three types. The equal distribution of muscle MUs' types was selected so that differences in force production between these three groups would be more distinct. It is visible from Figure 1(a), however, that the chosen 30 twitches were homogenously distributed. This is consistent with our previous study (Raikova et al. [30]) based on 114 experimentally measured twitches of MUs of the same muscle, where it was shown that the twitch parameters are distributed continuously and their values overlap for different MU types. The dependence of *F*_max⁡_ on *T*_*c*_ is also in agreement with the relationship given by Fuglevand et al. (see Figure 1(B) in Fuglevand et al. [6]), though not so strict. The equal proportion between *S*, FR, and FF MUs does not undermine the main conclusions in the paper since the distribution of the 6 parameters of the 30 twitches shown in Figure 1(a) is homogenous, and thus these 30 MUs closely match a population of MUs in real muscle. The main differences in these three groups do not concern the distribution of the twitches but rather other MU characteristics, namely, resistance to fatigue, sag appearance (i.e., decline in force after the initial increase during unfused tetanic stimulation), doublet presence, and so on, and these characteristics are not subject of the present study.

For each MU the software can generate individual firing patterns at a given mean frequency and its range. Therefore, the IPIs can be equal as in Figures 2, 4(a), and 4(c) or randomly generated as in Figures 4(e) and 4(g). Moreover, in Figure 5 the IPIs were generated using mean frequencies determined in relation to the MUs contraction times. So, for slow MUs we have simulated the mean firing rates in the range 24.2–40.0 Hz, whereas higher frequencies, namely, 42.1–59.7 Hz and 43.2–61.5 Hz, have been generated and used for FR and FF MUs, respectively. These frequencies correspond to the steep part of the force-frequency curves for particular MU types [38]. It is well known that during voluntary activity of MUs, the motoneuronal firing rates also correspond to this steep part of the force-frequency relationship [32, 39–42]. The modeled behaviour of MUs can also be compared to the MU firing properties of freely moving rats. Hennig and Lømo [32] recorded and analyzed EMG signals from rat soleus and extensor digitorum longus muscles (containing slow and fast MUs, resp.) and observed that their firing rates ranged 12–29 Hz in soleus and 40–111 Hz in extensor digitorum longus muscle. 

The model is open for further extension and implementation of new elements. The next step planned out is to solve the task opposite to the one presented in this paper, namely, to find the appropriate firing frequency impulsation of individual MUs necessary to reach a given muscle force. Of course, this is a highly indeterminate task. One solution has been proposed in Raikova and Aladjov [26], on the basis of a hierarchical genetic algorithm with which the impulses to each MU are chosen to fulfill a specific set of criteria. Another solution to this problem, based on correcting error between the target and the calculated force, has been proposed in the paper of Lowery and Erim [9]. Obviously, various types of feedbacks have to be taken into account [9, 43–45]. The present simulations show that there is a relationship between the time delay for recruitment of the next MUs and the increase in force (Figures 5(a) and 5(b)). The same refers to the derecruitment process. To model these relationships, it is possible to use the concept of one common “excitatory drive” for all MUs of a muscle to generate the firing pattern and respective values for the file *impulses.txt*. However, this relationship would be different for models with different MU populations, which makes the feedback concept highly important. Since the size principle is accepted as a general rule, the following scheme can be tested: first, the weakest slow MU is activated it starts to fire with an irregular pattern with a given mean frequency related to the contraction time. If the whole muscle force is lower than the expected one after a time increment Δ*t*, in moment *t*_*i*_, the next MU starts to fire, and this continues until the calculated and given force in *t*_*i*_ coincide. This procedure has to be performed for the moments *t*_*i*_ + *k*Δ*t* until the first maximum of the muscle force. Although this operation can work during recruitment, it will not work for modeling the derecruitment process. The reason is that a residual force remains from the active MUs, which is especially essential for slow MUs, having long relaxation periods. If the expected force decrease is fast, this residual force cannot be compensated only by deactivation of the active MUs. In reality, likely coactivation of antagonistic muscles is very important in reduction of the net joint torque [26]. Thus, if only one muscle is modelled, but not a complex of synergistic and antagonistic muscles, we would be unable to simulate all phenomena observed in real movements.

We have presented results from simulation of a simplified muscle model consisting of 30 MUs. There are few important observations that should also be introduced into modeling in the future to improve the degree of accuracy of the model so that it reflects real muscle. First, it is assumed that summation of the forces of MUs is linear, but it has been shown that this assumption is not always true and summation of MU forces is a nonlinear process. For the rat medial gastrocnemius muscle, when two MUs are stimulated in parallel, effects of summation of their forces can be either less than linear or more than linear, probably due to overlapping of MU territories [46]. It has been shown in the same study that after coactivation of several groups of MUs, the recorded force is regularly lower than the one expected on the basis of simple algebraic summation. The second important suggestion for the future concerns the variability of twitch responses to successive pulses within the unfused tetani. Our previous results obtained by a decomposition of tetanic curves of various MUs have shown that the experimental tetanic curve is considerably different from the one obtained by summation of equal twitches, and this difference is especially evident for slow MUs [37]. This difference can be avoided by using regression equations by which the parameters of the successive twitch-shape contractions within the tetanus are calculated using the level of the force at which the contraction starts [47]. It has been shown that not only the maximal twitch force has to be properly changed but all other twitch parameters as well. The third future task concerns the fact that in this paper we have chosen an equal number of MUs from the three main types—*S*, FR, and FF. In fact, different muscles are composed of different percentages of these three types of MUs [33, 48, 49]. This is determined in the input data in the file *datatw.txt*, and its contents can easily be changed. The data file can contain a real number and proportion of MUs in the modeled muscle. Experimental estimation for the male rat medial gastrocnemius indicates 8 *S*, 23 FR, and 26 FF MUs [50]. Fourth, to simulate longer activity of muscles, phenomena like force potentiation and fatigue should be taken into account by appropriately changing the parameters of the successive twitch-like contractions. Moreover, different phenomena concerning changes in firing rates of different MUs (including doublets and frequency increase of fast MUs due to fatigue) can easily be modeled by appropriate composition of the data in the text file *impulses.txt*.
